# Contact Compliance Based Visual Feedback for Tool Alignment in Robot Assisted Bone Drilling

**DOI:** 10.3390/s22093205

**Published:** 2022-04-21

**Authors:** Ping-Lang Yen, Yu-Jui Chen

**Affiliations:** Department of Biomechatronics Engineering, National Taiwan University, Taipei 10617, Taiwan; r07631026@ntu.edu.tw

**Keywords:** force control, human robot collaboration, surgical robot, medical robot

## Abstract

In recent decades, robot-assisted surgery has been proven superior at providing more accurate outcomes than the conventional one, particularly in minimally invasive procedures. However, there are still limitations to these kinds of surgical robots. Accurate bone drilling on the steep and hard surface of cortical bone is still challenging. The issues of slipping away from the target entry point on the bone surface and subsequently deviating from the desired path are still not completely solved. Therefore, in this paper, a force control is proposed to accompany the resolved motion rate controller in a handheld orthopedic robot system. The force control makes it possible to adjust the contact compliance of the drill to the bone surface. With the proper contact compliance, the drill can be prevented from deflecting in contact with the bone surface, and will eventually be directed to the target entry point. The experiments on test jig and vertebra phantom also show that the robot under the proposed contact compliance visual feedback control structure could produce better usability positioning accuracy under various contact disturbances than its counterpart.

## 1. Introduction

Bone drilling is common in orthopedic surgery. Patients suffering from bone fracture, trauma, and bone deformity are recommended to undergo surgical treatments [1]. These surgical procedures usually involve bone drilling to produce holes for screw insertion for fixation. For example, hip fractures or undisplaced intracapsular fractures are fixed with cannulated screws or dynamic hip screws [2]. Long bone fractures are fixed by intramedullary nails [3]. However, accurate bone drilling is often challenging, and can result in complications. Improper drilling, such as screw malalignment [4] and nail deformation during insertion [5,6] may result in poor fixation or damage to the surrounding tissues. In the pedicle screw fixation procedure, pedicle tunneling is critical for placing the pedicle screw. The accuracy of pedicle tunneling depends on the initial drilling entry point on the vertebra surface. Some of the target points are on the steep surfaces of vertebra. Drilling through the steep, slippery, and hard surface of cortical bone is demanding [7].

To address drill skidding, tool breakage, heat development and mechanical damage to surrounding tissues during bone drilling procedures, studies have been performed proposing various solutions ranging from drill design and computer navigation systems to robot assistance. A self-centering drill bit was designed to address low trauma requirements [8]. The drill structure allows drilling at the entrance and exit of bone with less skidding, less cutting force, and lower temperature. In recent decades, computer-assisted surgical systems, on the other hand, have adopted another approach by offering dynamic visualization of bones, the drilling tool, and implants to the surgeon for accurate alignment and fixation of bone fractures in minimally invasive orthopedic surgery [9]. This solution has been clinically proven to be more accurate than the conventional procedure, and thus able to reduce complications. However, computer-assisted surgery relies heavily on the surgeon’s proficiency in operating the handpiece. If the surgeon is too fatigued to steadily handle the handpiece, the drill could involuntarily deviate out of the desired path. Trying to keep the drill aligned with the desired path remains challenging and laborious. A handheld robot could provide an effective solution for alleviating the burden of manually preventing the tool from deviating [10]. One of the unsolved issues for the kind of robot-assisted drilling system is the tool skidding as the tool contacts the bone surface before breaking into the cortical bone surface. To address the related safety issue, several studies have focused on synthesizing the force sensing and control of the robot in order to improve bone drilling safety. For example, a cooperative robot was designed that integrated the real-time force sensing information into the bone drilling controller [11]. The interpretation of different bone drilling states during screw insertion into vertebra could provide information for the controller on whether the predefined critical safety conditions have been triggered. Lee et al. [12] similarly developed a drilling safety mechanism using the combined information of thrust force, drilling torque, and feed rate to automatically prevent tool movement in critical situations. Prediction of bone drilling conditions could be further enhanced by deep learning, so that the surgeon’s experience could be duplicated by a cybertwin model for better human–robot interaction performance [13,14]. Although the monitoring and trading control of the bone drill has been tackled, research taking into account the factors of tool deflection and slippage on the bone surface as the tool breaks through the bone surface are still lacking. Similar issues of tool deflection and runout occur in the traditional precision manufacturing sector when machining an inclined surface of metal. Precision manufacturing incorporates the prediction model for tool deflection and runout in order to reduce the dimensional errors [15,16]. In the robot-assisted bone drilling domain, the drilling accuracy can be similarly improved by deliberate monitoring of the tool alignment and the tool deflection. This paper addresses the above issue by proposing a controller that can steer and stabilize the drill bit to the desired entry point with less tool deflection. The contribution of this paper is to propose a force control together with a resolved motion rate control for a handheld robot to steer the tool towards the entry point by adjusting the contact compliance between the tool and the bone surface. The contact compliance adjustment was based on the tool deflection model and the measured force information. The proposed methodology was experimentally verified on a vertebra phantom with respect to entry point drilling accuracy because vertebra feature a steep and slippery surface at the entry points of the pedicles.

### 1.1. System Description of the Orthopedic Handheld Robot

**Figure 1:**
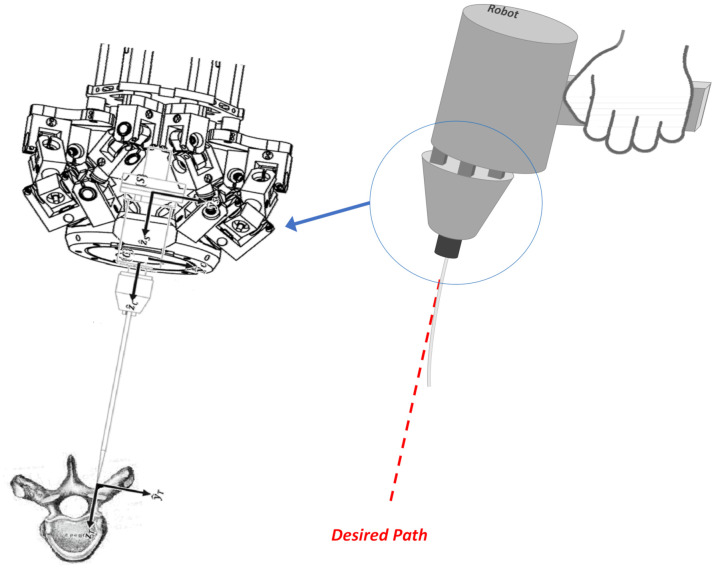
Schematic of a handheld robot with embedded force sensor.

The handheld robot shown in Figure 1 is a six-degree-of-freedom (DOF) parallel kinematic manipulator (PKM) designed for application in orthopedic surgery [17]. The end-effector is embedded with a six-axis force sensor (Nano, ATI, Pittsburgh, PA, USA) for measuring the forces/torques exerted on the drilling bit. 

**Figure 2:**
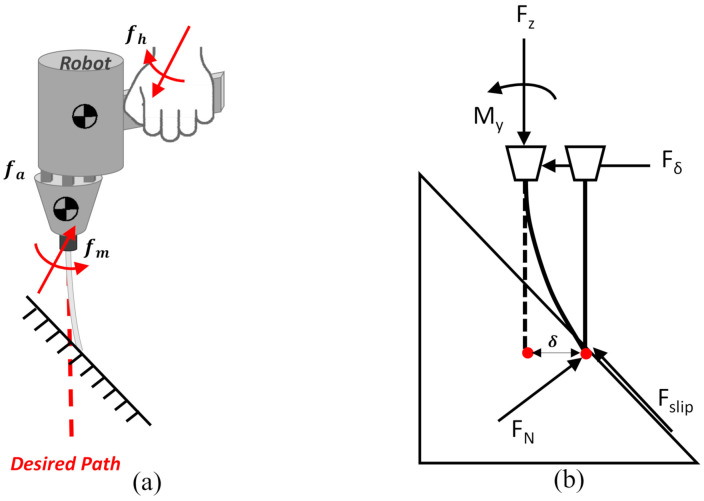
(**a**) The compensation action of the robot causes the drill to bend. At the same time, the deflection force of the drill tool is also reflected to the handle and felt by the operator, (**b**) the deflection model of the drill tool in contact with the object surface.

**Figure 3:**
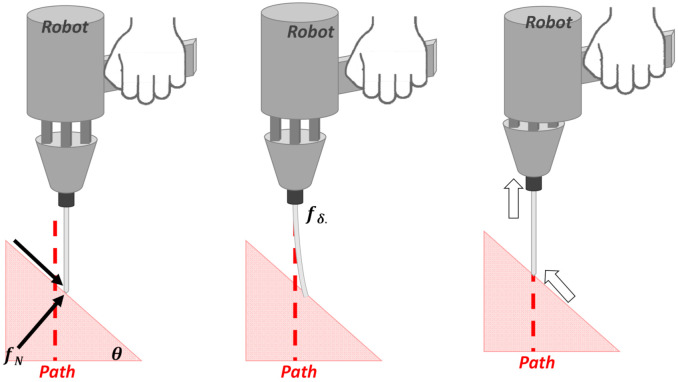
Deviation of the entry point caused by tool deflection when the handheld robot performs alignment correction for hand tremors.

As the drill approaches the surface of vertebra to which the drill is not perpendicular, the drill tends to skid away from the target drilling path. This is partially due to improper control of the exerted force with respect to the contact force situation. Too much pushing force on the tool when breaking the slippery and stiff bone surface usually produces tool skidding. The handheld robot is even more complicated, because the robot at the same time needs to stabilize the drill against disturbances caused by involuntary hand motion. In certain circumstances, the robot may cause tool deflection when moving the tool (Figure 2a) when the tool tip is still being maintained at the touch-down location. This situation provides the wrong information by the optical tracker regarding the actual location of the tool tip with respect to desired path because the tool is presumed to be rigid. This error can subsequently cause difficulties in correcting the deviation of the tool caused by the robot, because the tool is no longer un-deformed. In this case, a tool deflection model is necessary. Figure 2b shows the tool deflection model, which describes the forces acting on the bone surface originating from the drill and takes into account the deflection of the drill bit. Drill deflection indicates that the drill is likely unable to slide freely on the surface in order to correct the entry point (shown in Figure 3). In this case, contact compliance adjustment is necessary. During entry point correction, the actuators exert actuation forces in order to move the tool, while at the same time resulting in contact forces from the bone. Let {F} be the fiducial frame, {E} the effector frame, and {B} the handle frame; ***f_a_***, ***n_a_*** are the compensation force and torque from the actuators, then the dynamic equation of motion to describe the slave module (attaching the tool) expressed in {E} frame by Newton’s Euler method is:

(1) $$f_{m}=J_{r}f_{a}+M_{e}(a_{e}-g)+diag(k_{x},k_{y},k_{z})\delta \;$$

where $f_{d}$ and $n_{d}$ are measured by the force sensor; $r_{e}$ is the vector from the actuator to the center of mass of the end-effector module; $f_{a}$ is the output force vector of the six actuators; ***J_r_*** is the first three row vectors of the Jacobian matrix ***J*** of the PKM; M_e_ and is the mass matrix of the end-effector module; $a_{e}$ is the linear acceleration of the end-effector module, and can be calculated by measuring the frame {E} using the optical tracker; g is the gravitational acceleration. δ is the deflection displacement of the tool tip relative to the neutral axis of the tool. $k_{x}=k_{y}=$ $\frac{3EI}{L^{3}},$ where E is the Young’s modulus, L is the tool length, *I* is the moment of the inertia of the tool and $I=\frac{1}{4}\pi r^{4}$ where *r* is the radius of the tool, $k_{z}$ can be assigned as any value because $\delta_{z}$ is assumed to be zero. $f_{N}$ is the force normal to the neutral axis of the tool. The estimated deflection force is depicted as presented in Equation (2):

(2) $${\hat{f}}_{\delta }=f_{m}-J_{r}f_{a}+M_{e}(a_{e}-g)$$

where ${\hat{f}}_{\delta }$ is the estimated deflection force, calculated from the measured forces from the force sensor $f_{m}$, the actuation force $f_{a}$ in joint space, and the inertia force due to the acceleration $a_{e}$ of the end-effector. 

### 1.2. Control Strategy for Tool Self-Alignment

The force control adjusts the contact compliance, reduces the force normal to the bone surface, and enables the tool tip to slide along the surface for error correction of the entry point. This is achieved by adding a virtual spring to the tool using impedance force control. With the virtual spring, there are two advantages with respect to the contact force reduction: (1) the reduction in measurement error due to tool deformation and the effective compensation of tracking error; (2) the reduction in the reaction force resulting from the contact and its reflection to the human handle, thus preventing undesired deviation originating from the human hand. 

Let the modified equilibrium location $r_{d}^{c}$ was adjusted as Equation (3) by the spring constants $k_{x}$, $k_{y}$, $k_{z}$ of the virtual spring in X-, Y-, Z-directions. then we have:

(3) $$r_{d}^{c}=r_{d}-\left[\begin{array}{c} \begin{array}{c} k_{x}{\hat{f}}_{\delta x}\\ k_{y}{\hat{f}}_{\delta y}\\ k_{z}f_{N} \end{array}\\ \begin{array}{c} 0\\ 0\\ 0 \end{array} \end{array}\right]$$

The desired joint commands can be found from the resolved motion rate control [18], as presented in Equation (4):

(4) $${\dot{q}}_{d}=J^{-1}{\dot{r}}_{d}=J^{-1}K_{v}\left(r_{d}^{c}-r\right)=J^{-1}K_{v}\left(r_{d}-r\right)-J^{-1}K_{v}K\left[\begin{array}{c} \begin{array}{c} {\hat{f}}_{\delta x}\\ {\hat{f}}_{\delta y}\\ f_{N} \end{array}\\ \begin{array}{c} 0\\ 0\\ 0 \end{array} \end{array}\right]$$

where $K_{v}$ is the gain matrix for correcting the tracking error in Cartesian space. The gain matrix $K=diag\left(k_{x},\;k_{y},\;k_{z},\;0,0,0\right)$ is the stiffness control that is used to adjust the contact condition as the drill touches down the target surface.

### 1.3. Simulation

**Figure 4:**
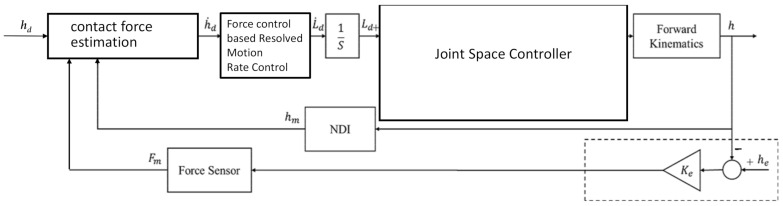
Block diagram for simulation.

The simulation model was configured as shown in Figure 4 by using the simulink^®^ software in MATLAB (Mathwork, Inc., Natick, MA, USA). The controller consists of three layers: force-controlled visual servo, resolved motion rate control, and joint space control. The forward kinematics of the PKM was constructed by a neural network model. The material of the tool was chosen a stainless steel #440, of which the Young’s modulus E=200 GP, the length was 120 mm, radius 1.5 mm. The elastic coefficient was calculated as $k_{tip}=1.38\;N/\mathrm{mm}$, which was also as the equivalent elastic coefficient $k_{eq}$ because the contact environment parameters *h_e_* and *K_e_* were assumed to be sufficiently rigid. The slope angle was set as θ=45°.

## 2. Experimental Setup

**Figure 5:**
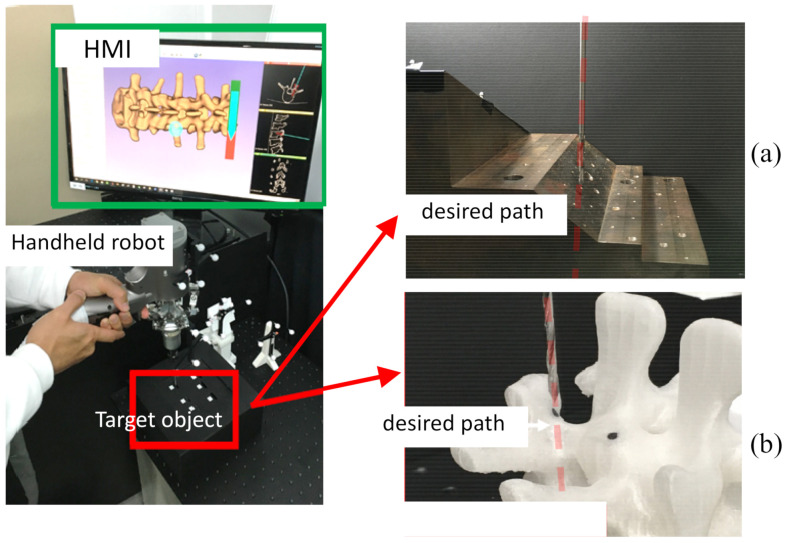
Experimental setup for drilling: (**a**) ASTM steel jig with constant slope, (**b**) vertebra phantom with variable slopes.

Two experiments were performed to validate the effectiveness of utilizing contact compliance adjustment for the accuracy improvement of entry point drilling when the desired path is not perpendicular to the contact surface. The first experiment was carried out using a testing jig with a constant slope of 45°, as shown in Figure 5a. The other one was performed on a vertebra phantom, the surface normal of which varied as shown in Figure 5b. These two phantoms were covered by a box to emulate the invisible scenario of the anatomy during minimally invasive surgery procedures. The operator only perceived the relative spatial information of the drill tip and the target path from the HMI created by 3D slicer software package [19]. The tactile feedback of the touching down force on the target surface was sensed by the operator directly from the handle of the handheld robot. The drill paths under resolved motion rate control with purely position-based control and in combination with force control were compared. In addition, the handle’s path and the contact force were also collected for analysis purposes.

## 3. Results and Discussions

### 3.1. Simulated Results

**Figure 6:**
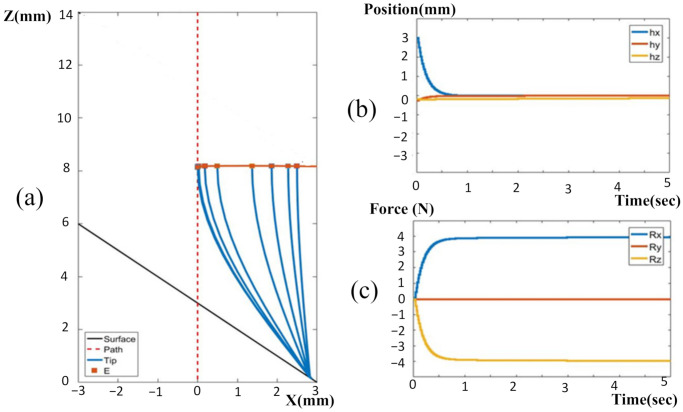
Adopting only position-based visual servo for entry point targeting: (**a**) tool tracking trajectory in XZ plane; (**b**) positioning errors in X-, Y- and Z-directions; (**c**) contact forces in X-, Y- and Z-directions.

The initial condition was set as a positioning error of 3 mm deviation from the target in the X-axis. Figure 6 shows the trajectory of the tool tip in the X–Z plane when only the position-based visual servo was applied to the resolved motion rate controller for entry point targeting. Although the end-effector moved to align the tool to the target point, the tool bent, and the tip was still anchored into the bone. 

**Figure 7:**
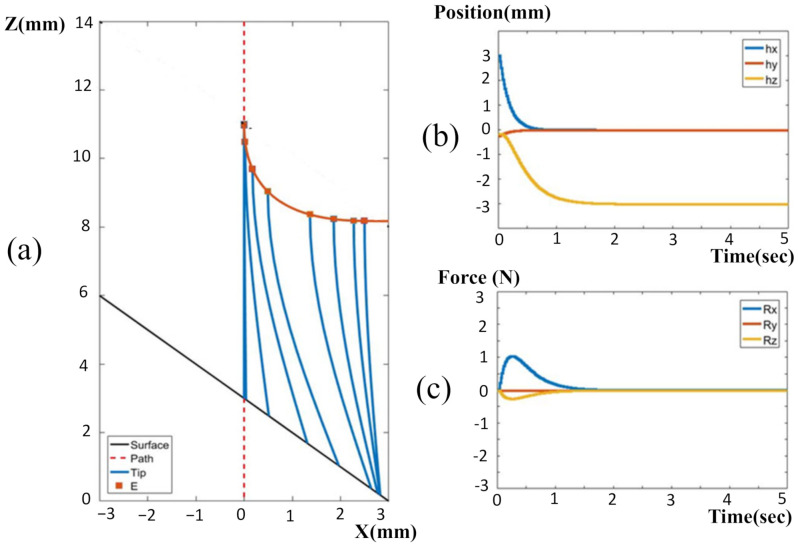
Contact compliance adjustment-based visual servo: (**a**) tool tracking trajectory in X-Z plane; (**b**) positioning errors in X-, Y- and Z-directions; (**c**) contact forces in X-, Y- and Z-directions.

Therefore, the tool still persisted under a position error with respect to the entry point, even though the end-effector had already compensated the measured error on the basis of the position sensor by the visual servo. At the same time, the measured forces in the X- and Z-directions display significant contact forces in these two directions. This could be used to predict the occurrence of a tool deflection using the elastic cantilever beam model. To utilize the deflection force information, the proposed force controller was used for the visual servo in order to calculate compensated commands for the resolved motion rate controller. The simulated results in Figure 7 indicate that the tool deflection could almost be eliminated by using contact compliance adjustment. As the contact forces (Figure 7c) were measured by the force sensor, the force controller could compute the corresponding compliance motion commands from the measured information for the visual servo to lift the tool in Z-axis direction while maintaining a certain amount of normal force in the tool. Subsequently, the tool deflection and deviation error could be alleviated. At the same time, the tool could slide toward the target entry point, leading to a reduction in the deviation error of the tool tip in the X-axis. Since the tool deflection has been alleviated, the contact force in the X-direction can eventually be reduced to very low level, depending on the friction force set in the simulation model.

The simulated results reveal several advantages of the force-controlled over the position-based only visual servo with respect to targeting the tool to the entry point: (1) Human involuntary motion causes the deviation of the tool from the targeted entry point. If this occurs, the position-based visual servo has limited ability to correct the error once the tool has been anchored to the object, because any correction motion by the robot may only cause the tool to be deflected instead of moving closer to the entry point. (2) The force-controlled visual servo can resolve the anchoring issue of the tool. The re-adjustment of the tool in the Z-axis allows the tool tip to be moved away from the anchor point and for the error to be corrected. The normal force is still maintained to assist in stabilizing the tool against sliding on the surface. (3) The force-controlled visual servo assists the tool in targeting the entry point through the local control of the tool tip without influencing the means of operation. 

### 3.2. Experiment Results

#### 3.2.1. Resolved Motion Rate without Contact Compliance Adjustment 

**Figure 8:**
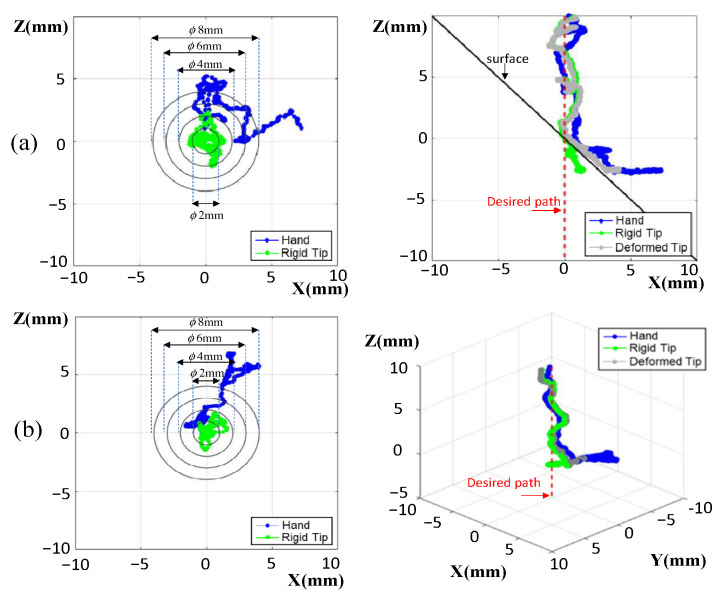
Tool trajectories upon approach to the target entry point without contact compliance adjustment: (**a**) on the ASTM test jig; (**b**) on the vertebra surface with varying slopes.

Figure 8 shows the trajectory of the drill under resolved motion rate control when the operator held the robot to target the entry point on an ASTM test jig. The slope of the jig was constant, at 45°. The coordinate system was defined, as the drilling direction was along the Z-axis, whereas the surface was parallel to the X-Y plane. Figure 8a shows the typical trajectory of the tool tip for the handheld robotic tool, where the tip under tremor suppression was still wandering by around 2 mm as it approached the drilling surface. At the time of the tip touching down on the surface, the tip was wandering less, since the tip was supported by the surface of the jig. At the touch down moment, the tip was with 1.5 mm away from the target entry point. The robot exerted a lateral force to pull/push the drill back into alignment with the desired path. This lateral force caused drill deformation, because the drill tip was anchored into the surface. The drill bit deformation was not shown in the navigation graphics. Instead, however, the HMI showed the tip (in green color) being moved back and aligned with the target path (red dash line), because the optical tracker only sensed the pose of the tool base. This may mislead the operator into starting to push the drill down to penetrate, resulting in further drill deflection or sliding on the surface. The reaction force was also reflected into the operator’s hand, with the operator sensing a lateral force to push away. This force artifact may produce confusion in the operator, or even lead to them removing the tool from the working space. Eventually the operator may have to pull up the tool and re-target the drill again. This implies that the handheld robot with only resolved motion rate control requires greater operator skill, and may also consume more time for drilling procedures. 

#### 3.2.2. Resolved Motion Rate with Contact Compliance Adjustment 

**Figure 9:**
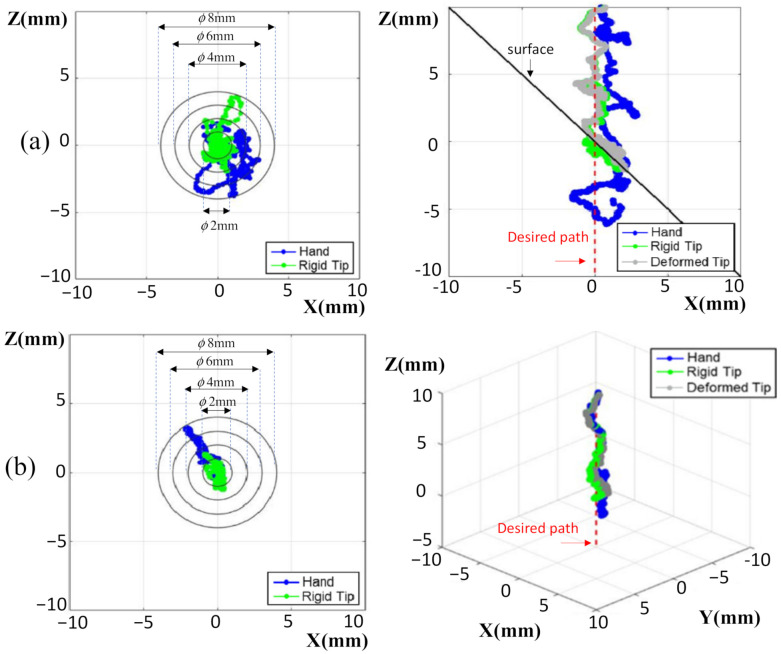
Tool trajectories upon approach to the target entry point with contact compliance adjustment: (**a**) on the ASTM test jig; (**b**) on the vertebra surface with varying slopes.

Figure 9 shows the results of drilling assisted by a handheld robot with force control. The force control assigns an impedance for the drill were proper contact to the surface is maintained, but sliding along the surface is still possible. On one hand, the surface can provide a support force for the drill to suppress involuntary hand motions. At the same time, the impedance control could still allow the resolved motion rate controller to correct the error of the drill tip. With contact compliance adjustment, drill deflection could be significantly reduced, and the tool trajectory shown on the navigation graphics was closer to the real pose of the drill tip. As the drill tip reaches the target entry point, the impedance can be switched to a higher value, and the operator informed to start to penetrate the surface. This also displays the advantage that the operator can point the tool easily without reaction force disturbance due to smaller reflected forces. Thus, the total consumed time for entry point targeting can be effectively reduced.

Table 1 summarizes the reaction forces and the position errors when targeting the tool tip to the entry point. The average reaction forces on the tool tip were more than 30% larger in the case without contact compliance adjustment compared to the case with contact compliance adjustment. In particular, the forces in the normal direction were reduced by the contact compliance adjustment, so that the tool could remain in proper contact condition to the surface while allowing the tool to slip across the surface to approach the target entry point. The forces reflected to the human operator were also reduced by contact compliance adjustment, and thus the handle wandering could be observed to decrease from 4.4 mm and 6.1 mm to 2.3 mm and 2.8 mm for the steel phantom and vertebra phantom, respectively. The results indicate that the robot with contact compliance adjustment could alleviate the force artifacts of tool deflection, and improve operational usability for the operator. The tool tip position errors (mean value) were also reduced from 1.1 mm to 0.8 mm for the ASTM test jig and from 0.7 mm to 0.6 mm for the vertebra phantom. The force control was demonstrated superiority in targeting the desired entry point under various contact disturbances. 

## 4. Conclusions

Material machining is very common in surgery as well as in the manufacturing industry [20]. Optimization of tool design and machining parameters for drilling associated with materials such as metal or bone is critical for achieving a better outcome. Although both share similar approaches, safety, in particular, is one of the most critical factors in surgical procedures. Therefore, the involvement of a human in the control loop is still the most acceptable structure among the current surgical robot systems. The kind of surgical robotic system featured in this article leverages both human perception and a robot’s accurate response during the bone drilling procedure. This paper demonstrated a human–robot collaboration for bone drilling by a handheld surgical robot system. In the handheld robot system, the embedded PKM could direct the mounted drill tool to align with the desired target path by compensating involuntary motion from the human hand. At the same time, the controller incorporated the tool contact compliance adjustment provided by the force control with the tool optical tracking provided by the resolved motion rate. As a result, the embedded PKM of the handheld robot was able to correct positioning errors with an appropriate contact compliance and to move the drill tool towards the entry point. The force control in the handheld robot control system makes it possible to increase the positioning accuracy for human–robot collaboration, in particular when the drill tool comes into contact with the anatomy surface. For future work, an artificial intelligence algorithm could be applied in order to learn from experienced surgeons [21] for further tactile recognition of the contact status when drilling various bones.

## Figures and Tables

**Table 1:** Comparison of the contact forces and position errors of the tool under resolved motion rate control with/without contact compliance adjustment.

Control Strategy	Phantom	Lateral Force *f_δ_*	Normal Force *f_N_*	Position Error of the Tool Tip	Position Error of the Handle
without contact compliance adjustment	constant slope	1.45 N	1.34 N	1.1 mm	4.4 mm
	varying slopes	1.08 N	2.18 N	0.7 mm	6.1 mm
with contact compliance adjustment	constant slope	0.75 N	0.87 N	0.8 mm	2.3 mm
	varying slopes	0.84 N	0.82 N	0.6 mm	2.8 mm
